# Micro-RNA Binding Site Polymorphisms in the WFS1 Gene Are Risk Factors of Diabetes Mellitus

**DOI:** 10.1371/journal.pone.0139519

**Published:** 2015-10-01

**Authors:** Zsuzsanna Elek, Nóra Németh, Géza Nagy, Helga Németh, Anikó Somogyi, Nóra Hosszufalusi, Mária Sasvári-Székely, Zsolt Rónai

**Affiliations:** 1 Department of Medical Chemistry, Molecular Biology and Pathobiochemistry, Semmelweis University, Budapest, Hungary; 2 2nd Department of Internal Medicine, Semmelweis University, Budapest, Hungary; 3 Research Laboratory, 3rd Department of Internal Medicine, Semmelweis University, Budapest, Hungary; Academia Sinica, TAIWAN

## Abstract

The absolute or relative lack of insulin is the key factor in the pathogenesis of diabetes mellitus. Although the connection between loss of function mutations of the *WFS1* gene and DIDMOAD-syndrome including diabetes mellitus underpins the significance of wolframin in the pathogenesis, exact role of *WFS1* polymorphic variants in the development of type 1 and type 2 diabetes has not been discovered yet. In this analysis, 787 patients with diabetes and 900 healthy people participated. Genotyping of the 7 *WFS1* SNPs was carried out by TaqMan assays. Association study was performed by *χ* ^2^-test in combination with correction for multiple testing. For functional analysis, the entire 3’ UTR of the *WFS1* gene was subcloned in a pMIR-Report plasmid and relative luciferase activities were determined. Linkage disequilibrium analysis showed a generally high LD within the investigated region, however the rs1046322 locus was not in LD with the other SNPs. The two miR-SNPs, rs1046322 and rs9457 showed significant association with T1DM and T2DM, respectively. Haplotype analysis also confirmed the association between the 3’ UTR loci and both disease types. *In vitro* experiments showed that miR-185 reduces the amount of the resulting protein, and rs9457 miRSNP significantly influences the rate of reduction in a luciferase reporter assay. Genetic variants of the *WFS1* gene might contribute to the genetic risk of T1DM and T2DM. Furthermore demonstrating the effect of rs9457 in binding of miR-185, we suggest that the optimal level of wolframin protein, potentially influenced by miR-regulation, is crucial in normal beta cell function.

## Introduction

Diabetes mellitus is a group of pathogenically heterogeneous diseases sharing the trait of absolute or relative insufficiency of insulin effect. Common forms of the disease are type 1 (T1DM) and type 2 diabetes mellitus (T2DM). T1DM results from autoimmune *β*-cell destruction leading to insulin deficiency, whereas type 2 diabetes (T2DM) is the end point of a progressive insulin secretion defect on the background of insulin resistance. Both T1DM and T2DM are considered to be complex diseases caused by multiple environmental and genetic risk factors. Polymorphisms and mutations of genes associated with other, much rarer monogenic forms of diabetes have been in the focus of research for many years. One of these illnesses is the Wolfram- or DIDMOAD-syndrome, characterized by diabetes insipidus, non-autoimmune and non-HLA-linked insulin dependent diabetes mellitus together with progressive degeneration of beta cells[1], optic atrophy and deafness[2]. The mutated gene causing the disease was denominated *WFS1* and is located on chromosome 4 region p16[2]. It was suggested that polymorphisms of the *WFS1* resulting in minor modulation of the gene function instead of complete loss might be in the genetic background of the common, polygenic forms of diabetes (T1DM and T2DM). Meta-analysis of association studies revealed that the rs1046320 and rs10010131 SNPs were significant risk factors of T2DM[3]. The two SNPs were in strong linkage disequilibrium with each other, and although no biological effect could be demonstrated, it is notable that the rs1046320 polymorphism is located in the 3’ UTR of the *WFS1* gene.

Significance of the miRNA system in the fine regulation of protein synthesis has recently been discovered. Although the principle of miRNA-action is their binding to the 3’ UTR of target genes, prediction of this interaction based on solely sequence alignment is doubtful. 355 miRNAs were suggested to have a binding site in the *WFS1* 3’ UTR *in silico* by the miRWalk database[4], however this interaction could be confirmed by molecular methods only for miR-21 and the members of the hsa-let-7 family. Similarly 11439 genes (including *WFS1*) were predicted to be the putative target of miR-185, whereas only 33 (excluding *WFS1*) have been confirmed *in vitro* so far. Considerable data suggest that miRNAs have a direct role in insulin secretion and production, pancreatic islet development, insulin action and indirectly control glucose and lipid metabolism[5]. The miRWalk database suggested a connection between diabetes mellitus and 140 miRNAs. Although miR-185 was not among these hits, it was demonstrated by a recent study that both miR-185 and miR-668 were expressed in pancreatic islets[6]. Moreover, analyzing numerous tissues it was observed that miR-185 was abundant, high expression level was detected in the brain, kidney, lung, placenta, prostate, spleen and thyroid glands[7]. On the other hand miR-668 showed an islet-specific expression[6]. Our earlier study[8] revealed that miR-668 not only bound to the 3’ UTR of the *WFS1* gene, but this connection was influenced by the rs1046322 SNP, which showed a significant association with diabetes mellitus according to our current findings (Tables 1, 2, 3 and 4).

**Table 1:** Allele-wise association analysis of the investigated *WFS1* SNPs and diabetes mellitus.

	*T1DM*						*T2DM*						*DM*					
SNP, Risk allele	Freq. T1DM	Freq. Control	p	OR	Lower CI	Upper CI	Freq. T2DM	Freq. Control	p	OR	Lower CI	Upper CI	Freq. DM	Freq. Control	p	OR	Lower CI	Upper CI
**rs6824720**, G	0.841	0.812	0.145	1.221	0.933	1.597	0.875	0.824	**0.004**	1.505	1.137	1.990	0.858	0.824	**0.027**	1.293	1.029	1.624
**rs10010131**, G	0.639	0.605	0.134	1.154	0.957	1.393	0.689	0.609	**2.5∙10** ^**−4**^	1.425	1.178	1.722	0.664	0.608	**0.002**	1.270	1.090	1.480
**rs13147655**, G	0.649	0.599	0.052	1.233	0.998	1.523	0.689	0.606	**4.5∙10** ^**−4**^	1.444	1.176	1.774	0.669	0.606	**0.002**	1.317	1.108	1.566
**rs1801208**, G	0.959	0.958	0.883	1.039	0.627	1.720	0.956	0.950	0.550	1.151	0.725	1.828	0.958	0.950	0.352	1.204	0.814	1.782
**rs1046320**, A	0.598	0.589	0.675	1.038	0.871	1.237	0.674	0.592	**1.1∙10** ^**−4**^	1.429	1.191	1.714	0.635	0.591	**0.011**	1.205	1.044	1.390
**rs1046322**, A	0.187	0.090	**3.9∙10** ^**−11**^	2.327	1.802	3.004	0.139	0.094	**0.001**	1.555	1.191	2.032	0.163	0.094	**5.9∙10** ^**−9**^	1.887	1.520	2.343
**rs9457**, C	0.601	0.544	**0.008**	1.264	1.062	1.504	0.631	0.546	**8.1∙10** ^**−5**^	1.421	1.193	1.698	0.616	0.546	**5.1∙10** ^**−5**^	1.334	1.160	1.533

Tests were carried out using an allele-wise approach. p: *p* value of statistical significance of the *χ* ^2^-probe. Bold numbers show nominally significant results, underlined *p* values are significant using FDR approach, whereas double underlined numbers mean significant results after Bonferroni correction for multiple testing. Freq.: allele frequencies, OR, Lower CI, Upper CI: Odds-ratio with 95% upper and lower confidence intervals. Analyses were carried out in both disease types (T1DM: 2*N* = 814, control: 2*N* = 1634; T2DM: 2*N* = 760, control: 2*N* = 1784) as well as in the combined patient group (DM: 2*N* = 1574, control: 2*N* = 1800).

**Table 2:** Association analysis of the investigated *WFS1* SNPs and diabetes mellitus by Cochran–Armitage trend test.

SNP	*T1DM*	*T2DM*	*DM*
rs6824720	0.173	**0.006**	**0.038**
rs10010131	0.121	**1.8∙10** ^**−4**^	**0.002**
rs13147655	0.064	**7.1∙10** ^**−4**^	**0.003**
rs1801208	0.932	0.551	0.372
rs1046320	0.726	**2.2∙10** ^**−4**^	**0.020**
rs1046322	**2.6∙10** ^**−8**^	**0.003**	**9.2∙10** ^**−7**^
rs9457	**0.031**	**8.7∙10** ^**−4**^	**8.5∙10** ^**−4**^

The table depicts the *p* values of statistical significance. Bold numbers show nominally significant results, underlined *p* values are significant using FDR approach, whereas double underlined numbers mean significant results after Bonferroni correction for multiple testing. Analyses were carried out in both disease types (T1DM: *N* = 407, control: *N* = 817; T2DM: *N* = 380, control: *N* = 892) as well as in the combined patient group (DM: *N* = 787, control: *N* = 900).

**Table 3:** Genotype-wise association analysis of the investigated *WFS1* SNPs and diabetes mellitus using recessive model.

	*T1DM*						*T2DM*						*DM*					
SNP, Risk genotype	Freq. T1DM	Freq. Control	p	OR	Lower CI	Upper CI	Freq. T2DM	Freq. Control	p	OR	Lower CI	Upper CI	Freq. DM	Freq. Control	p	OR	Lower CI	Upper CI
**rs6824720**, GG	0.726	0.671	0.104	1.297	0.947	1.775	0.777	0.687	**0.004**	1.591	1.157	2.189	0.751	0.687	**0.017**	1.376	1.058	1.790
**rs10010131**, GG	0.430	0.366	**0.046**	1.305	1.004	1.696	0.488	0.367	**1.4∙10** ^**−4**^	1.645	1.272	2.126	0.459	0.366	**3.5∙10** ^**−4**^	1.472	1.190	1.820
**rs13147655**, GG	0.450	0.377	**0.043**	1.354	1.009	1.817	0.484	0.373	**0.001**	1.575	1.190	2.084	0.467	0.373	**0.002**	1.473	1.158	1.875
**rs1801208**, GG	0.919	0.919	0.988	1.004	0.597	1.688	0.912	0.904	0.692	1.102	0.683	1.777	0.915	0.904	0.489	1.155	0.768	1.735
**rs1046320**, AA	0.388	0.352	0.224	1.169	0.909	1.503	0.465	0.351	**1.8∙10** ^**−4**^	1.609	1.254	2.064	0.426	0.350	**0.002**	1.376	1.124	1.685
**rs1046322**, AA	0.086	0.012	**7.0∙10** ^**−10**^	7.641	3.608	16.186	0.030	0.011	**0.023**	2.699	1.109	6.571	0.058	0.011	**3.1∙10** ^**−7**^	5.465	2.645	11.290
**rs9457**, CC	0.375	0.303	**0.013**	1.377	1.068	1.776	0.420	0.304	**7.5∙10** ^**−5**^	1.654	1.288	2.125	0.397	0.304	**8.5∙10** ^**−5**^	1.505	1.227	1.846

Table shows the frequency values of homozygotes for the risk allele (recessive model). p: *p* values of statistical significance of the *χ* ^2^-probe. Bold numbers show nominally significant results, underlined *p* values are significant using FDR approach, whereas double underlined numbers mean significant results after Bonferroni correction for multiple testing. Freq.: allele frequencies, OR, Lower CI, Upper CI: Odds-ratio with 95% upper and lower confidence intervals. Analyses were carried out in both disease types (T1DM: *N* = 407, control: *N* = 817; T2DM: *N* = 380, control: *N* = 892) as well as in the combined patient group (DM: *N* = 787, control: *N* = 900).

**Table 4:** Genotype-wise association analysis of the investigated *WFS1* SNPs and diabetes mellitus using dominant model.

	*T1DM*						*T2DM*						*DM*					
SNP, Risk genotypes	Freq. T1DM	Freq. Control	p	OR	Lower CI	Upper CI	Freq. T2DM	Freq. Control	p	OR	Lower CI	Upper CI	Freq. DM	Freq. Control	p	OR	Lower CI	Upper CI
**rs6824720**, GG+AG	0.956	0.953	0.866	1.061	0.533	2.111	0.973	0.960	0.304	1.513	0.684	3.345	0.964	0.960	0.709	1.125	0.606	2.089
**rs10010131**, GG+AG	0.847	0.843	0.875	1.029	0.721	1.468	0.891	0.851	0.073	1.425	0.966	2.101	0.869	0.851	0.347	1.155	0.856	1.558
**rs13147655**, GG+AG	0.847	0.822	0.362	1.199	0.811	1.770	0.895	0.838	**0.019**	1.638	1.080	2.486	0.871	0.838	0.115	1.306	0.937	1.821
**rs1801208**, GG+AG	1.000	0.997	0.301	1.003	0.997	1.008	1.000	0.995	0.190	1.005	0.998	1.011	1.000	0.995	0.060	1.005	0.998	1.005
**rs1046320**, AA+AG	0.807	0.825	0.445	1.129	0.826	1.544	0.883	0.832	**0.023**	1.525	1.059	2.196	0.844	0.831	0.490	1.099	0.841	1.435
**rs1046322**, AA+AG	0.289	0.168	**2.7∙10** ^**−6**^	2.011	1.497	2.700	0.248	0.177	**0.004**	1.537	1.142	2.069	0.268	0.176	**1.1∙10** ^**−5**^	1.717	1.347	2.188
**rs9457**, CC+CG	0.828	0.785	0.081	1.319	0.966	1.802	0.842	0.787	**0.026**	1.437	1.043	1.979	0.835	0.787	**0.015**	1.364	1.062	1.751

Table shows the frequency values of homo- and heterozygous carriers for the risk allele (dominant model). p: *p* values of statistical significance of the *χ* ^2^-probe. Bold numbers show nominally significant results, underlined *p* values are significant using FDR approach, whereas doubled underlined numbers mean significant results after Bonferroni correction for multiple testing. Freq.: allele frequencies, OR, Lower CI, Upper CI: Odds-ratio with 95% upper and lower confidence intervals. Analyses were carried out in both disease types (T1DM: *N* = 407, control: *N* = 817; T2DM: *N* = 380, control: *N* = 892) as well as in the combined patient group (DM: *N* = 787, control: *N* = 900).

In this study we demonstrate an association between both types (T1DM and T2DM) of diabetes mellitus and SNPs in the *WFS1* gene. Three of the investigated polymorphisms (rs1046320 A/G, rs1046322 A/G and rs9457 C/G) are located in the 3’ UTR of the gene, rs1801208 A/G is in exon 8, whereas rs6824720 A/G, rs10010131 A/G and rs13147655 A/G are in intron 1, 4 and 5 respectively. We show that besides the rs1046322 locus, described earlier[8], the rs9457 SNP also influences miRNA binding in an *in vitro* system. These data might help in elucidating the molecular background of the disease and might contribute to the elaboration of novel, alternative therapeutic approaches.

## Materials and Methods

### Participants, DNA-purification

407 patients diagnosed with T1DM (from the 3^rd^ Department of Internal Medicine, Szentágothai Knowledge Center, Semmelweis University, 46.9% female, 53.1% male, disease onset at the age of 32.1 ± 10.5 years) and 380 patients with T2DM (from the 2^nd^ Department of Internal Medicine at the Semmelweis University, 57.9% female, 42.1% male, disease onset at the age of 48.0 ± 12.7 years) participated in the study. The 900 healthy volunteers (59.0% female, 41.0% male, mean age: 39.0 ± 13.1 years) were recruited by the Institute of Psychology, Eötvös Loránd University. The study protocol was approved by the Local Ethical Committee (ETT-TUKEB ad.328/KO/2005, ad.323-86/2005-1018EKU from the Scientific and Research Ethics Committee of the Medical Research Council). To address the issue of population stratification, the participation was restricted to subjects of Hungarian origin, thus creating an ethnically homogenous population. All participants signed written informed consent before providing DNA sample for genetic testing. Buccal epithelial cells were collected by swabs in duplicates (two swabs per sample and two samples per person). DNA purification was initiated by the incubation of the buccal samples at 56°C overnight in 0.2 mg/ml Proteinase K cell lysis buffer. It was followed by denaturing the proteins with saturated NaCl solution. Finally, DNA was precipitated using isopropanol and subsequently by 70% ethanol. DNA pellet was resuspended in 100 μL 0.5× TE (1× TE: 10 mM Tris pH = 8.0; 1 mM EDTA) buffer. Concentration of the DNA-samples was measured by NanoDrop1000 spectrophotometer.

### SNP Genotyping

Seven SNPs in the *WFS1* gene were genotyped by using pre-designed TaqMan assays from Life Technologies (rs6824720: C__30903802_10; rs10010131: C__30473796_10; rs13147655: C__22272143_10; rs1801208: C___2401737_10; rs1046320: C___2873369_10; rs1046322: C___8841086_1_; rs9457: C___2873371_10). These assays contained the two primers and allele-specific fluorescent probes labeled by FAM and VIC, respectively. Reaction mixtures contained these primer–probe premixes, the TaqMan^®^ Genotyping Master Mix (including AmpliTaq Gold^®^ DNA-polymerase, deoxyribonucloside-triphosphates and buffer) according to manufacturer’s instructions as well as approximately 4 ng genomic DNA in a final volume of 6 μL. A 7300 Real Time PCR instrument was used for amplification. Thermocycle was started by activating the hot start DNA-polymerase and denaturing genomic DNA at 95°C for 10 minutes. This was followed by 40 cycles of denaturation at 95°C for 15 sec, and combined annealing and extension at 60°C for 1 minute. Real-time detection was carried out during the latter step for higher sensitivity and reliability to verify the results of the subsequent post-PCR plate read and automatic genotype call.

Genotype data in “ped” format are available as S1–S4 Tables.

### Plasmid construction

The full length 3’ untranslated region (3’ UTR) of the *WFS1* gene was amplified by PCR using a DNA-sample of an individual with known rs9457 (homozygous CC) genotype. The following primers were used: forward primer: 5’ TCG GCG GAG CTC GGA TGG TCC GCC ACG AGG AGC 3’ (where the *Sac* I site is underlined) and reverse primer: 5’ AAA GGA AAG CTT GCG CTG CAG GTT CCA CCA GAG G 3’ (where the *Hin*d III site is underlined). The PCR products were digested with *Sac* I and *Hin*d III and were cloned downstream of the firefly luciferase gene in pMIR-Report plasmid (pMIR-REPORT miRNA Expression Reporter Vector System; ABI) by T4 DNA ligase (New England BioLabs) using standard protocols. The generated reporter construct (denoted as C) carried the C (risk) allele, the other one (denoted as G) carrying the protective G allele was generated by Quick Change Lightning (Stratagene) site directed mutagenesis. A reference construct (referred to as Seed) contained a completely modified seed region: site-directed mutagenesis was employed to modify the appropriate segment of this construct. A control construct contained a different DNA-insert with the same length lacking any sequence complementary to miR-185 (denoted as Control). All constructs were confirmed by DNA sequencing.

### MiRNA binding assay

Human Embryonic Kidney cell line (HEK293T) was cultured in DMEM medium (Invitrogen—Gibco), supplemented with 10% bovine fetal serum (Lonza). The cells were maintained at 37°C in an atmosphere of 5% CO_2_. For miRNA assays the cells were seeded into 24-well plates and incubated for 24 hours before transfection. Subsequently, 0.05 μg of the luciferase reporter construct were cotransfected with 0.2 μg *β*-galactosidase plasmid (Ambion) and 5 pmol of miR185 and/or miR668 (Sigma-Aldrich) using Lipofectamine 2000 (Invitrogen) according to the manufacturer’s instruction. When investigating the effect of the presence or absence of miR185 and miR668, miR20b was used as supplement to use the same amount of total miRNA in each sample to avoid any spurious effect. Cells were collected 48 hours after transfection, washed with PBS, then extracted by three consecutive freeze–thaw cycles and subsequent centrifugation. Supernatants were used for enzyme activity measurements. Luciferase activity was measured by adding 60 μl Luciferin reagent (0.16 mg/ml Luciferin K, 20 nM Tricine, 2.6 nM MgSO_4_, 0.1 nM Na_2_EDTA, 33.3 nM DTT, 0.27 nM Li_3_CoA and 0.53 nM Na_2_ATP) to 12 μl of each cell extract. Luminescence was measured using a Varioskan multi-well plate reader (Thermo Fisher Scientific, Inc.). Values for luciferase activity were normalized to *β*-galactosidase activity (measured by standard protocol using the same Varioskan plate reader in photometry mode). Each experiment was independently repeated three times, and each sample was studied in triplicate.

### Statistical analysis

The Hardy–Weinberg equilibrium (HWE) for genotype distributions was assessed by *χ* ^2^-test. No significant difference (*p* > 0.1) could be observed between the measured and expected genotype frequencies in the control group for any of the investigated SNPs. The frequency of both T1DM and T2DM is similar in females and males, and both disease types can develop at any age, although T1DM is more characteristic in juveniles, whereas symptoms of T2DM usually appear in patients older than 40. Despite, age- and sex-matched study design was used in each analysis to rule out any spurious association. Allele-wise association analysis and genotype-wise tests using dominant and recessive models were carried out by comparing the allele or genotype frequency values of each polymorphism in the patient and control group using SPSS v17.0 and HaploView v4.2[9]. GWASpi[10] was applied to perform Cochran–Armitage trend test[11]. Correction for multiple testing was carried out to avoid false positive results using two different approaches. False Discovery Rate (FDR) was calculated by Microsoft Excel, results were verified using a web-based tool (http://www.sdmproject.com/utilities/?show=FDR) [12]. The more stringent Bonferroni correction was also applied, the accepted level of statistical significance was 0.05 / 84 = 5.95 · 10^−4^, as 7 loci and 3 patient categories (T1DM, T2DM and combined) were analyzed in 4 different approaches. Haplotype analysis was carried out by HaploView v4.2[9]. The same software package was used to linkage disequilibrium analysis. ANOVA of the luciferase assays was carried out using GraphPad InStat (version 3.05).

## Results

### Marker selection

Seven SNPs of the *WFS1* gene were recruited in our study. Three (rs1046320, rs1046322 and rs9457) of them were located in the 3’ UTR of the gene. The rs1046322 and rs9457 polymorphisms were suggested to be miR-SNPs altering the binding site of the miR-668 and miR-185 respectively by the Patrocles[13] and PolymiRTS[14] databases. Moreover, putative regulatory effect of the rs1046322 site has previously been demonstrated by *in vitro* luciferase reporter system[8]. The rs1046320 SNP was shown to be in association with type 2 diabetes mellitus, however no biological function could be detected[3]. The rs687420, rs10010131 and rs13147655 polymorphisms are intronic variants, whereas rs1801208 is located in the last exon of the gene. These sites have been selected based on literature data, and our earlier thorough SNP analysis of the *WFS1* gene including 17 polymorphic loci[8] recruiting at least one polymorphism from each haplotype block.

### Linkage disequilibrium analysis

**Fig 1:**
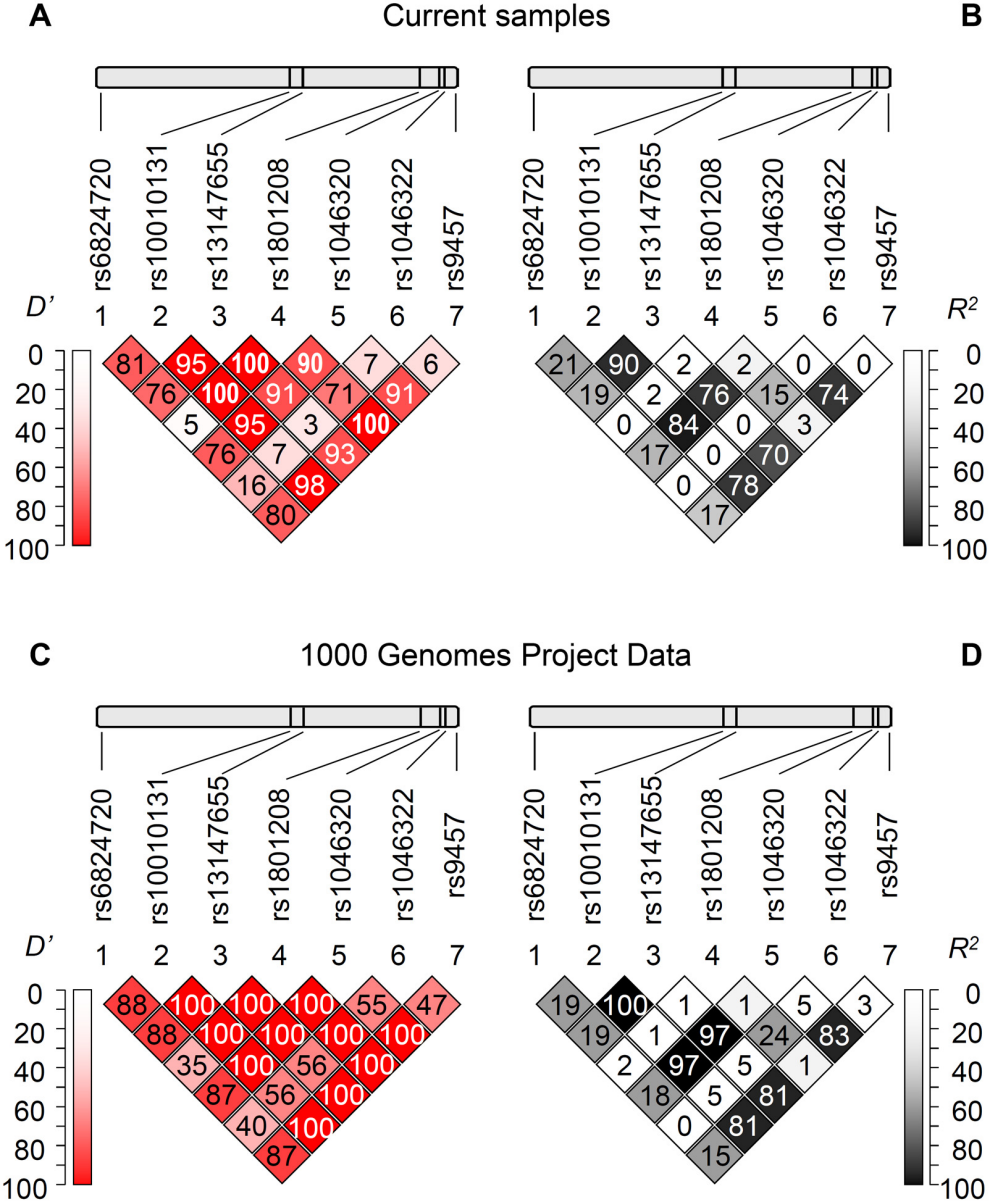
Linkage disequilibrium analysis of the investigated *WFS1* SNPs. Upper panels indicate the chromosomal positions of the polymorphisms, below the pairwise linkage disequilibrium data of the SNPs are demonstrated. Lewontin’s *D’* (Panels A and C) and *R* ^2^ values (Panels B and D) calculated based on our genotype data (Panels A and B) as well as based on the data obtained from the 1000 Genomes Project (Panels C and D) are shown. Dark background and *D’* or *R* ^*2*^ = 100 values mean strong linkage disequilibrium between the two appropriate loci. (Colored version of the figure is available online.)

Fig 1 Panel A and B show the pairwise linkage disequilibrium (LD) analysis based on our population including healthy control subjects and patients demonstrating Lewontin’s *D’* and *R* ^2^ values, respectively. These results were in good agreement with the LD analysis using the data set of the 1000 Genomes Project (Fig 1 Panel C and D). Generally a high level of LD can be observed in the gene region, although it is notable that the rs1046322 polymorphism was not in linkage disequilibrium with the other sites. Moreover the high *D’* values in combination with the low *R* ^2^ values regarding the rs1801208 polymorphism show a partial linkage disequilibrium of this locus with other SNPs, demonstrating the occurrence of three and the lack of one haplotype out of the four theoretical pairwise allele combinations.

### Association studies

Table 1 summarizes the results of the case-control study in an allele-wise setting. Analyses were carried out for both diabetes types as well as for the combined patient group using age- and sex-matched controls. Interestingly the risk allele was the same for the type 1 and the type 2 forms of the disease, and it is also remarkable, that it was only the rs1046322 SNP, at which this variant was the minor allele. It is also notable that polymorphisms in rather different locations of the gene associated with diabetes mellitus, although no complete linkage disequilibrium could be shown in this region. However the lowest *p* values of statistical significance could be detected in case of two 3’ UTR SNPs. The rs1046322 and rs9457 polymorphisms showed nominally significant (*p* < 0.05) association in all three (type 1, type 2 and combined) analyses, which remained significant in all cases after FDR correction for multiple testing. The association between the rs1046322 polymorphism and T1DM as well as that between rs9457 SNP and T2DM could be declared even using the more stringent Bonferroni approach (accepted level of significance: *p* ≤ 5.95 · 10^−4^). Moreover, the rs10010131, the rs13147655 and the rs1046320 SNPs were also found to significantly associate with type 2 diabetes even using FDR or Bonferroni correction.

The Cochran–Armitage trend test was employed to extend the association study in a genotype-wise manner (Table 2). In agreement with the allele-wise test, no association could be detected between the rs1046320 locus and T1DM (*p* = 0.726). The major effect of the rs1046322 SNP in T1DM was also confirmed (*p* = 2.639 · 10^−8^), whereas a weak nominal association (*p* = 0.031) could be observed between rs9457 locus and the type 1 disease form. The Cochran–Armitage trend test detected association in the T2DM group in case of 6 SNPs, all of them remained significant using FDR correction. The allele- and genotype-wise analyses were in good agreement in the combined diabetes group as well. Strong association could be detected in case of two 3’ UTR polymorphisms (rs1046322 and rs9457), the former one remained significant even using Bonferroni correction.

To further analyze association between the 7 WFS1 SNPs and diabetes mellitus in a more detailed and systematic way, genotype-wise tests were carried out applying two different approaches (Tables 3 and 4). Frequency values of the genotype consisting of both risk alleles were computed for all loci in each patient group using the recessive model. In this setup a remarkably high odds ratio (7.641) was observed in case of the rs1046322 SNP in the T1DM disease group, whereas the significant association between rs10010131, rs1046320, rs9457 polymorphisms and T2DM was also confirmed. When using the dominant model, i.e. comparing the frequency of risk allele carriers and noncarriers, the effects detected previously could be observed. Association was found between the rs1046322 locus and T1DM, as well as between all three 3’ UTR SNPs and the rs13147655 polymorphism and type 2 disease form using FDR correction, however none of these results remained significant by the stringent Bonferroni method. This observation shows that the effect of the risk allele is more pronounced in homozygous form suggesting that the recessive model is more appropriate in this case, however it can also be remarked that pure recessive or dominant inheritance can only be expected in case of monogenic diseases.

Based on the putative synergistic effect of the three 3’ UTR SNPs (rs1046320, rs1046322 and rs9457) in translational regulation, these polymorphisms were included in a haplotype-wise association study. Investigation of the patient group with type 1 diabetes mellitus confirmed the major effect of the rs1046322 SNP. Frequency of the haplotypes containing the risk (“A”) allele of this site in combination with the risk variant of one or both SNPs (Table 5) was significantly higher in the patient group. This resulted in an outstanding odds ratio value, which was above 3 in all cases. Similar leader effect of a single polymorphism could not be observed in case of type 2 diabetes. The “AC” haplotype of the rs1046320 and rs9457 SNPs showed a highly significant association with the disease, on the other hand odds ratio was the lowest in case of this combination, and it is also remarkable that these two polymorphisms are in linkage disequilibrium with each other, which can also contribute to the observed low statistical *p* value of the *χ* ^2^-probe.

**Table 5:** Haplotype analysis of the 3’ UTR SNPs of the *WFS1* gene.

	*T1DM*						*T2DM*						*DM*					
Haplotype, risk allel	Freq. T1DM	Freq. Control	p	OR	Lower CI	Upper CI	Freq. T2DM	Freq. Control	p	OR	Lower CI	Upper CI	Freq. DM	Freq. Control	p	OR	Lower CI	Upper CI
**rs1046322–rs9457**, AC	0.124	0.042	**3.4∙10** ^**−14**^	3.262	2.369	4.492	0.067	0.038	**0.001**	1.843	1.267	2.681	0.098	0.043	**2.9∙10** ^**−10**^	2.412	1.821	3.194
**rs1046320–rs9457**, AC	0.548	0.542	0.790	1.023	0.864	1.211	0.626	0.555	**0.001**	1.341	1.127	1.596	0.583	0.542	**0.017**	1.181	1.030	1.354
**rs1046320–rs1046322**, AA	0.124	0.043	**1.1∙10** ^**−13**^	3.165	2.304	4.347	0.070	0.040	**0.001**	1.809	1.254	2.609	0.100	0.045	**4.0∙10** ^**−10**^	2.368	1.795	3.124
**rs1046320–rs1046322–rs9457**, AAC	0.120	0.042	**2.8∙10** ^**−13**^	3.152	2.285	4.348	0.066	0.038	**0.002**	1.805	1.238	2.630	0.093	0.043	**6.1∙10** ^**−9**^	2.274	1.713	3.019

p: *p* value of statistical significance of the association between the investigated haplotype and T1DM (2*N* = 814, control: 2*N* = 1634) or T2DM (2*N* = 760, control: 2*N* = 1784) or the combined patient group (DM: 2*N* = 1574, control: 2*N* = 1800) assessed by *χ* ^2^-analysis. Bold numbers mean statistically significant association. Freq.: allele frequencies, OR, Lower CI, Upper CI: Odds-ratio with 95% upper and lower confidence intervals.

### *In vitro* miRNA binding assay

**Fig 2:**
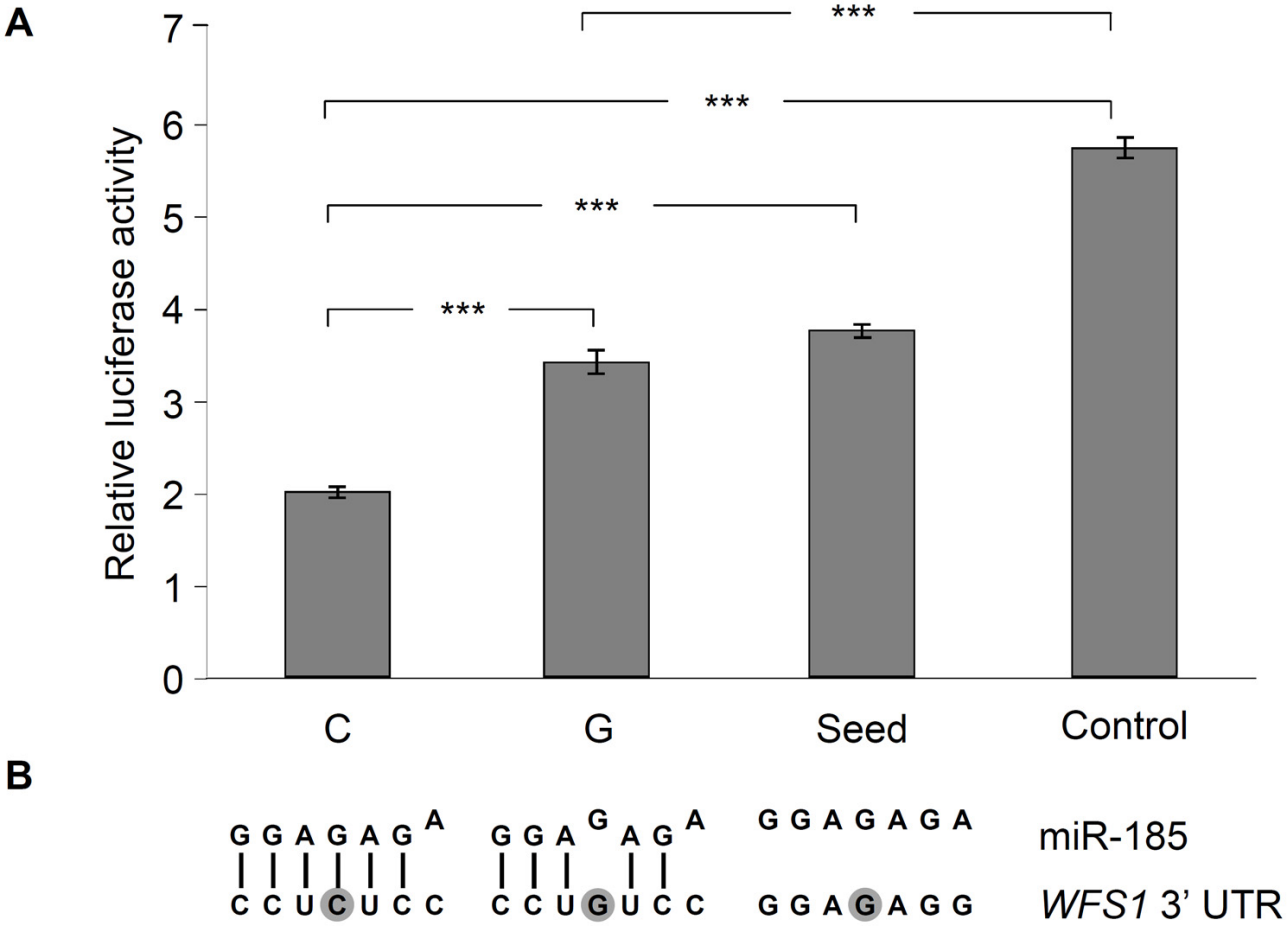
Analysis of the effect of *WFS1* rs9457 SNP on miR-185 binding by luciferase reporter assay. The entire 3’ UTR region of the *WFS1* gene was subcloned into the pMIR luciferase riporter vector. Transient transfection were performed in HEK293 cells, for details, see Method section. Luciferase activity values normalized to *β*-galactosidase activity were measured, *Panel A* represents average ± SD values of three independent experiments. (*** p < 0.001). The “C” luciferase construct harbored the rs9457 C allele, whereas the “G” contained the rs9457 G variant. “Seed” construct was generated by changing all seven nucleotides in the core binding site of WFS1 3’ UTR region. “Control” construct contained a different DNA-insert with the same length lacking any sequence complementary to miR-185. *Panel B* demonstrates the sequence alignment of miR-185 and its binding site in the *WFS1* 3’ UTR. Mismatches are shown by the shifted nucleotides of the miRNA, the rs9457 locus is shown by dark background.

Putative role of the rs9457 locus on miRNA binding was analyzed by *in vitro* luciferase assay, as according to the prediction of the PolymiRTS[14] database, this SNP is located in the binding site of the seed sequence of microRNA-185 (miR-185). The 3’ UTR region of the *WFS1* gene was subcloned in a pMIR-Report luciferase reporter vector, construct with the “G” allele at the rs9457 locus was created by site directed mutagenesis. The same approach was used to generate the “seed mutant” construct, which lacked the entire binding site of the seed sequence of miR-185. Moreover a construct without any binding site of miR-185 (referred to as “Control”) was also used. Luciferase enzyme levels were normalized to *β*-galactosidase activities. The lowest relative luciferase activity (35% of the “Control”) could be measured with the construct containing a “C” allele of the rs9457 polymorphism (Fig 2). Interestingly a single mismatch caused by the SNP resulted in a more, than 1.7 times increase (*p* < 0.05) in luciferase level, and this value was practically identical with the activity of the “seed” construct. It might be related to the finding of the *in silico* sequence alignment, which showed only 6 complementary bases out of the 7 nucletotides of the seed sequence in case of the “C” allele, and the “G” variant adds a further mismatch into the region (Fig 2, Panel B), resulting in a hypothetically even weaker interaction between miRNA and mRNA. The putative role of miRNA-based regulation was further analyzed by investigating the haplotype containing the rs1046320 “A”–rs1046322 “A”–rs9457 “C” variants, which are the risk alleles of diabetes (Table 5) according to our association study. Earlier we demonstrated that the rs1046322 locus altered the binding efficiency of miR-668[8], now the combined effect of miR-185 and miR-668 was assessed. Relative luciferase activities were measured in the presence or absence of any or both of miR-185 and miR-668, respectively. Presence of both miRNAs resulted in a 0.71 relative luciferase activity (*p* = 0.013) compared to the reference sample (without miR-668 and miR-185). Application of only miR-185 caused a somewhat smaller effect (relative activity: 0.78, *p* = 0.036). On the other hand miR-668 did not result in a significant decrease (relative activity: 0.89, *p* = 0.251) in relative luciferase level. This observation might be explained by the fact that the rs1046320 “A” allele leads to a mismatch in the binding site of the seed region of miR-668, thus its weaker binding and milder effect can be expected.

## Discussion

### Role of wolframin in the development of diabetes mellitus

Relationship between wolframin and diabetes mellitus has been suggested at numerous levels. Although the *WFS1* gene is abundant, high expression level was detected in pancreatic islets[2] and in insulinoma *β*-cell lines[15]. The 100 kDa wolframin protein possesses 9 transmembrane polypeptide regions and is located in the membrane of the endoplasmic reticulum (ER), the N-terminal of the protein is oriented in the cytoplasm, whereas the C-terminal is in the lumen of the ER. Its structure and localization suggest that the protein might play a role in membrane transport and might contribute to the Ca^2+^ homeostasis of the ER[16]. This was confirmed by the observation of[17] that wolframin expression increases the Ca^2+^ concentration of the cytosol in Xenopus oocytes. Connection between ER homeostasis and diabetes mellitus is well known. The increased insulin production caused by insulin resistance in T2DM leads to chronic ER stress and this contributes to the death of *β*-cells by apoptosis. It was demonstrated that glucose induced insulin secretion leads to increased WFS1 expression in wild type mice, whereas ER stress and *β*-cell dysfunction can be observed in *WFS1* knock-out animals[18].

Our data further support the connection between wolframin and diabetes mellitus at a genetic level. Loss of function mutations are well known causes of the monogenic DIDMOAD syndrome[2], as well as some SNPs of the gene have been shown to be in association with T2DM[3]. Our data confirm the association between the gene and the disease, moreover extend this knowledge in some aspects. First, it is important to note that our study included a large group of both T1DM and T2DM patients and we detected association with both disease types. The rs1046322 SNP was found to be a strong risk factor of T1DM and showed a weaker association with T2DM as well. The rs9457 SNP was demonstrated to be in stronger association with the type 2 form of the disease, and the association between rs10010131 and T2DM[3] was confirmed. Haplotype analysis revealed the significance of the 3’ UTR polymorphisms in both forms of the illness. Although the symptoms and clinical appearance of T1DM and T2DM might be rather different, this observation is in agreement with the accelerator hypothesis of Wilkin[19] suggesting that patomechanism and some molecular dysfunctions overlap in the two disease types. On the other hand our results suggested and supported molecular and functional connection between the identified SNPs and the disease. Our *in vitro* molecular assays demonstrated that the rs1046322[8] and the rs9457 SNPs are located in miRNA binding sites and might alter the binding efficiency of the corresponding miRNA. This effect might play a role *in vivo* resulting in slightly different wolframin levels in case of the presence of different allele and haplotype patterns.

### MiRNA regulation

Numerous evidences suggest, that the impaired regulation of wolframin level can be one of the molecular mechanisms leading to diabetes. MiRNAs are important components of this regulatory network, and the multiple connection between miRNAs and the disease has recently been demonstrated[20]. MiRNAs were shown to influence ADP: ATP ratio[21, 22], exocytosis of insulin granules[23] and insulin synthesis[24]. MiRNAs were also suggested to contribute to the development of insulin resistance through the protein kinase B / GLUT 4 translocation pathway[25–27]. Altered expression of miRNAs was observed in both T1DM[28] and T2DM[29] including miR-185, which was shown to influence *β*-cell activity by targeting the mRNA of suppressor of cytokine signaling 3 (SOCS3)[30]. This is observation underlines the fact that the miRNA regulation network is rather complex. There are several miRNAs that influence the translation of the same target. It is notable that we earlier described an *in vitro* interaction between *WFS1* 3’ UTR and miR-668[8], and demonstrated in the current project that the rs1046322 SNP influencing the affinity of miR-668 to *WFS1* mRNA showed a strong association with diabetes mellitus. It was also observed that the WFS1 3’ UTR with the risk haplotype (rs1046320 “A”–rs1046322 “A”–rs9457 “C”) is sensitive to both miR-185 and miR-668, although the effect of miR-185 seemed to be stronger in the *in vitro* luciferase assay. Moreover, one miRNA can be the regulator of numerous mRNAs. The data of Bao[30] and our results show, that these different pathways can be in the background of the same phenotype, as miR-185 is suggested to be related to diabetes mellitus via two different (*SOCS3* and *WFS1*) targets.

Taking these data together, interaction of miRNAs and *WFS1* could be of great importance in the molecular background of diabetes mellitus. This is further supported by a recent comprehensive study focusing on the miRNA profile of pancreatic *β*-cells[6]. Comparison of the mRNA targets of islet specific miRNAs and candidate genes of T2DM based on genome-wide association studies resulted in six hits including *WFS1* underlining the putative *in vivo* relevance of the findings of our results obtained by *in vitro* luciferase assays.

### Linkage disequilibrium study

Linkage disequilibrium analysis is an important supplement of association studies in several aspects. On one hand, loci in LD might help to identify candidate genes or chromosome regions associating with a phenotype, on the other hand although tag SNPs show statistical association, despite they are often not the polymorphisms that possess biological effect. Our earlier study analyzed 17 SNPs of the *WFS1* gene in another sample set[8], these results were used for SNP selection in this study. LD analysis of the 7 polymorphisms in our current work was in good agreement not only with the results of our earlier findings, but also with the LD analysis of the genotype data available from 1000 Genomes Project. These results suggest two observations. First, pairwise *D’* and *R* ^2^ values of the rs1046322 SNP with other loci are low suggesting that this SNP is not a genetic marker only, but presumably possesses direct biological effect. This is in good agreement with our earlier observations[8] demonstrating the effect of rs1046322 SNP on the alteration on binding efficiency of miR-668 *in vitro*, and it is notable that this miR was shown to be specific for pancreatic islets[6]. It is also in concordance with our data suggesting the functional role of rs9457 polymorphism in miR-185 binding, which miR shows a high expression level in islet cells, and these observations support the importance of miR-based regulation of wolframin level in the molecular background of diabetes. On the other hand, rs9457 SNP is in LD with several other loci, such as rs1046320. This could clarify the results of an earlier study[3] demonstrating an association of the rs1046320 SNP with diabetes, however failing to show any functional effect of this locus. It might be explained by our findings: as the two sites are in linkage disequilibrium, the rs1046320 polymorphism can be considered to be a genetic marker of the rs9457 site, which possesses a molecular function.

In conclusion, our study not only confirms that polymorphic variants of the *WFS1* gene are the genetic components of T2DM but also suggests loci that might play a molecular role in the development of the illness. Moreover, we demonstrated that these SNPs can also contribute to the development of T1DM form of the disease. Besides, we showed association between novel 3’ polymorphisms and diabetes mellitus, these loci were demonstrated to be miR-SNPs altering the binding efficiency of miR-185 and miR-668. These results might contribute to the understanding of molecular processes in the background of diabetes mellitus, which could be of clinical significance providing the bases of novel approaches of therapy or secondary prevention.

## Supporting Information

S1 Table SNP information file: “s1_dm.info”. This file contains the required information about SNPs necessary for the analysis of “.ped” files.
(INFO)

S2 Table Genotype data for the association analysis in the T1DM group: “s2_t1dm.ped”. This file contains the genotype data of the T1DM patients and its control group.
(PED)

S3 Table Genotype data for the association analysis in the T2DM group: “s3_t2dm.ped”. This file contains the genotype data of the T2DM patients and its control group.
(PED)

S4 Table Genotype data for the association analysis in the combined group: “s4_dm.ped”. This file contains the genotype data of the combined group of patients with either type of diabetes mellitus and the control samples.
(PED)

## References

[1] Ganie MA, Bhat D. Current developments in Wolfram syndrome. Journal of pediatric endocrinology & metabolism: JPEM. 2009;22(1):3–10. Epub 2009/04/07. . 19344068 10.1515/jpem.2009.22.1.3

[2] Inoue H, Tanizawa Y, Wasson J, Behn P, Kalidas K, Bernal-Mizrachi E, et al A gene encoding a transmembrane protein is mutated in patients with diabetes mellitus and optic atrophy (Wolfram syndrome). Nat Genet. 1998;20(2):143–8. Epub 1998/10/15. 10.1038/2441 . 9771706

[3] Fawcett KA, Wheeler E, Morris AP, Ricketts SL, Hallmans G, Rolandsson O, et al Detailed investigation of the role of common and low-frequency WFS1 variants in type 2 diabetes risk. Diabetes. 2010;59(3):741–6. Epub 2009/12/24. 10.2337/db09-0920 20028947 PMC2828659

[4] Dweep H, Sticht C, Pandey P, Gretz N. miRWalk—database: prediction of possible miRNA binding sites by "walking" the genes of three genomes. Journal of biomedical informatics. 2011;44(5):839–47. Epub 2011/05/25. 10.1016/j.jbi.2011.05.002 . 21605702

[5] Dehwah MA, Xu A, Huang Q. MicroRNAs and type 2 diabetes/obesity. Journal of genetics and genomics = Yi chuan xue bao. 2012;39(1):11–8. Epub 2012/02/02. 10.1016/j.jgg.2011.11.007 . 22293113

[6] van de Bunt M, Gaulton KJ, Parts L, Moran I, Johnson PR, Lindgren CM, et al The miRNA profile of human pancreatic islets and beta-cells and relationship to type 2 diabetes pathogenesis. Plos One. 2013;8(1):e55272 Epub 2013/02/02. 10.1371/journal.pone.0055272 PONE-D-12-26003 [pii]. 23372846 PMC3555946

[7] Landgraf P, Rusu M, Sheridan R, Sewer A, Iovino N, Aravin A, et al A mammalian microRNA expression atlas based on small RNA library sequencing. Cell. 2007;129(7):1401–14. 10.1016/j.cell.2007.04.040 ISI:000247911400024. 17604727 PMC2681231

[8] Kovacs-Nagy R, Elek Z, Szekely A, Nanasi T, Sasvari-Szekely M, Ronai Z. Association of aggression with a novel microRNA binding site polymorphism in the wolframin gene. American journal of medical genetics Part B, Neuropsychiatric genetics: the official publication of the International Society of Psychiatric Genetics. 2013;162B(4):404–12. Epub 2013/05/08. 10.1002/ajmg.b.32157 . 23650218

[9] Barrett JC, Fry B, Maller J, Daly MJ. Haploview: analysis and visualization of LD and haplotype maps. Bioinformatics. 2005;21(2):263–5. Epub 2004/08/07. 10.1093/bioinformatics/bth457 . 15297300

[10] Muniz-Fernandez F, Carreno-Torres A, Morcillo-Suarez C, Navarro A. Genome-wide association studies pipeline (GWASpi): a desktop application for genome-wide SNP analysis and management. Bioinformatics. 2011;27(13):1871–2. Epub 2011/05/19. 10.1093/bioinformatics/btr301 . 21586520

[11] Clarke GM, Anderson CA, Pettersson FH, Cardon LR, Morris AP, Zondervan KT. Basic statistical analysis in genetic case-control studies. Nature protocols. 2011;6(2):121–33. Epub 2011/02/05. 10.1038/nprot.2010.182 21293453 PMC3154648

[12] Benjamini Y, Drai D, Elmer G, Kafkafi N, Golani I. Controlling the false discovery rate in behavior genetics research. Behavioural brain research. 2001;125(1–2):279–84. Epub 2001/10/30. . 11682119 10.1016/s0166-4328(01)00297-2

[13] Hiard S, Charlier C, Coppieters W, Georges M, Baurain D. Patrocles: a database of polymorphic miRNA-mediated gene regulation in vertebrates. Nucleic acids research. 2010;38(Database issue):D640–51. Epub 2009/11/13. 10.1093/nar/gkp926 19906729 PMC2808989

[14] Bao L, Zhou M, Wu L, Lu L, Goldowitz D, Williams RW, et al PolymiRTS Database: linking polymorphisms in microRNA target sites with complex traits. Nucleic acids research. 2007;35(Database issue):D51–4. Epub 2006/11/14. 10.1093/nar/gkl797 17099235 PMC1669716

[15] Hofmann S, Philbrook C, Gerbitz KD, Bauer MF. Wolfram syndrome: structural and functional analyses of mutant and wild-type wolframin, the WFS1 gene product. Hum Mol Genet. 2003;12(16):2003–12. Epub 2003/08/13. . 12913071 10.1093/hmg/ddg214

[16] Strom TM, Hortnagel K, Hofmann S, Gekeler F, Scharfe C, Rabl W, et al Diabetes insipidus, diabetes mellitus, optic atrophy and deafness (DIDMOAD) caused by mutations in a novel gene (wolframin) coding for a predicted transmembrane protein. Hum Mol Genet. 1998;7(13):2021–8. Epub 1998/11/18. doi: ddb264 [pii]. . 9817917 10.1093/hmg/7.13.2021

[17] Osman AA, Saito M, Makepeace C, Permutt MA, Schlesinger P, Mueckler M. Wolframin expression induces novel ion channel activity in endoplasmic reticulum membranes and increases intracellular calcium. J Biol Chem. 2003;278(52):52755–62. Epub 2003/10/07. 10.1074/jbc.M310331200 . 14527944

[18] Fonseca SG, Fukuma M, Lipson KL, Nguyen LX, Allen JR, Oka Y, et al WFS1 is a novel component of the unfolded protein response and maintains homeostasis of the endoplasmic reticulum in pancreatic beta-cells. J Biol Chem. 2005;280(47):39609–15. Epub 2005/10/01. 10.1074/jbc.M507426200 . 16195229

[19] Wilkin TJ. Diabetes: 1 and 2, or one and the same? Progress with the accelerator hypothesis. Pediatric diabetes. 2008;9(3 Pt 2):23–32. Epub 2008/06/11. 10.1111/j.1399-5448.2007.00343.x . 18540866

[20] Chen H, Lan HY, Roukos DH, Cho WC. Application of microRNAs in diabetes mellitus. The Journal of endocrinology. 2014;222(1):R1–R10. Epub 2014/05/02. 10.1530/JOE-13-0544 . 24781254

[21] Pullen TJ, da Silva Xavier G, Kelsey G, Rutter GA. miR-29a and miR-29b contribute to pancreatic beta-cell-specific silencing of monocarboxylate transporter 1 (Mct1). Molecular and cellular biology. 2011;31(15):3182–94. Epub 2011/06/08. 10.1128/MCB.01433-10 21646425 PMC3147603

[22] Ramachandran D, Roy U, Garg S, Ghosh S, Pathak S, Kolthur-Seetharam U. Sirt1 and mir-9 expression is regulated during glucose-stimulated insulin secretion in pancreatic beta-islets. The FEBS journal. 2011;278(7):1167–74. Epub 2011/02/04. 10.1111/j.1742-4658.2011.08042.x . 21288303

[23] Roggli E, Gattesco S, Caille D, Briet C, Boitard C, Meda P, et al Changes in microRNA expression contribute to pancreatic beta-cell dysfunction in prediabetic NOD mice. Diabetes. 2012;61(7):1742–51. Epub 2012/04/28. 10.2337/db11-1086 22537941 PMC3379668

[24] Zhao X, Mohan R, Ozcan S, Tang X. MicroRNA-30d induces insulin transcription factor MafA and insulin production by targeting mitogen-activated protein 4 kinase 4 (MAP4K4) in pancreatic beta-cells. J Biol Chem. 2012;287(37):31155–64. Epub 2012/06/27. 10.1074/jbc.M112.362632 22733810 PMC3438947

[25] Meerson A, Traurig M, Ossowski V, Fleming JM, Mullins M, Baier LJ. Human adipose microRNA-221 is upregulated in obesity and affects fat metabolism downstream of leptin and TNF-alpha. Diabetologia. 2013;56(9):1971–9. Epub 2013/06/13. 10.1007/s00125-013-2950-9 23756832 PMC3737431

[26] Chen YH, Heneidi S, Lee JM, Layman LC, Stepp DW, Gamboa GM, et al miRNA-93 inhibits GLUT4 and is overexpressed in adipose tissue of polycystic ovary syndrome patients and women with insulin resistance. Diabetes. 2013;62(7):2278–86. Epub 2013/03/16. 10.2337/db12-0963 23493574 PMC3712080

[27] Lee H, Jee Y, Hong K, Hwang GS, Chun KH. MicroRNA-494, upregulated by tumor necrosis factor-alpha, desensitizes insulin effect in C2C12 muscle cells. Plos One. 2013;8(12):e83471 Epub 2013/12/19. 10.1371/journal.pone.0083471 24349514 PMC3859653

[28] Salas-Perez F, Codner E, Valencia E, Pizarro C, Carrasco E, Perez-Bravo F. MicroRNAs miR-21a and miR-93 are down regulated in peripheral blood mononuclear cells (PBMCs) from patients with type 1 diabetes. Immunobiology. 2013;218(5):733–7. Epub 2012/09/25. 10.1016/j.imbio.2012.08.276 . 22999472

[29] Zampetaki A, Kiechl S, Drozdov I, Willeit P, Mayr U, Prokopi M, et al Plasma microRNA profiling reveals loss of endothelial miR-126 and other microRNAs in type 2 diabetes. Circulation research. 2010;107(6):810–7. Epub 2010/07/24. 10.1161/CIRCRESAHA.110.226357 . 20651284

[30] Bao L, Fu X, Si M, Wang Y, Ma R, Ren X, et al MicroRNA-185 targets SOCS3 to inhibit beta-cell dysfunction in diabetes. Plos One. 2015;10(2):e0116067 Epub 2015/02/07. 10.1371/journal.pone.0116067 25658748 PMC4319748

